# Active Fragment of *Veronica ciliata* Fisch. Attenuates t-BHP-Induced Oxidative Stress Injury in HepG2 Cells through Antioxidant and Antiapoptosis Activities

**DOI:** 10.1155/2017/4727151

**Published:** 2017-11-22

**Authors:** Yiran Sun, Qiuxia Lu, Libo He, Yueyue Shu, Shiyan Zhang, Shancai Tan, Lin Tang

**Affiliations:** ^1^Key Laboratory of Bio-Resources and Eco-Environment of Ministry of Education, College of Life Sciences, Sichuan University, Chengdu, Sichuan 610065, China; ^2^National and Local Joint Engineering Laboratory for Energy Plant Bio-Oil Production and Application, Chengdu, Sichuan 610065, China

## Abstract

Excessive amounts of reactive oxygen species (ROS) in the body are a key factor in the development of hepatopathies such as hepatitis. The aim of this study was to assess the antioxidation effect in vitro and hepatoprotective activity of the active fragment of *Veronica ciliata* Fisch. (VCAF). Antioxidant assays (DPPH, superoxide, and hydroxyl radicals scavenging) were conducted, and hepatoprotective effects through the application of *tert*-butyl hydroperoxide- (t-BHP-) induced oxidative stress injury in HepG2 cells were evaluated. VCAF had high phenolic and flavonoid contents and strong antioxidant activity. From the perspective of hepatoprotection, VCAF exhibited a significant protective effect on t-BHP-induced HepG2 cell injury, as indicated by reductions in cytotoxicity and the levels of ROS, 8-hydroxydeoxyguanosine (8-OHdG), and protein carbonyls. Further study demonstrated that VCAF attenuated the apoptosis of t-BHP-treated HepG2 cells by suppressing the activation of caspase-3 and caspase-8. Moreover, it significantly decreased the levels of ALT and AST, increased the activities of acetyl cholinesterase (AChE), glutathione (GSH), superoxide dismutase (SOD), and catalase (CAT), and increased total antioxidative capability (T-AOC). Collectively, we concluded that VCAF may be a considerable candidate for protecting against liver injury owing to its excellent antioxidant and antiapoptosis properties.

## 1. Introduction

Overloaded oxidative stress generated by reactive oxygen species (ROS), including the superoxide anion, hydroxyl, and H_2_O_2_, may promote disease development and progression through the oxidative damage of biological molecules such as proteins, lipids, and DNA [1-3]. Oxidative stress is one of the primary factors in the development of hepatopathy, including hepatitis and cirrhosis [4, 5]. To combat liver failure caused by oxidative stress, the body produces several antioxidases that elevate antioxidant activity, such as superoxide dismutase (SOD), catalase (CAT), and glutathione (GSH). Cellular antioxidant enzymes and the free radical scavengers protect cells from the oxidative stress effects of the ROS [1, 6, 7].

Many studies have reported that medicinal plants that possess bioactive components, such as flavonoids and phenolic acids, are effective for the prevention of oxidative stress-related liver pathologies by either direct action on ROS or indirect effects on the antioxidant defense system. Therefore, they have been suggested as an alternative to the development of new drugs for the applied treatment of many diseases, such as hepatopathy. [8, 9].

*Veronica ciliata* Fisch., a member of the Scrophulariaceae family, is a traditional Tibetan medicine used in more than 100 types of Tibetan medicine prescriptions, particularly for hepatoprotection, which contributes to its remarkable effects on cholecystitis, hepatitis, urticaria, and rheumatism [10]. Polyphenols and iridoid glycosides isolated from *V. ciliata* Fisch. are the main active compounds and have been reported to exert multiple effects, such as antioxidant, anticancer, anti-inflammatory, and hepatoprotective activities [11, 12, 13]. Previous studies have shown that ethyl acetate extracts of *V. ciliata* Fisch. exhibited stronger antioxidant and hepatoprotective activities than those exhibited by the aqueous extracts of *V. ciliata* Fisch. against carbon tetrachloride- and acetaminophen- (unpublished) induced liver injury in mice [14]. Further investigations indicated that the iridoid glycoside fraction isolated from *V. ciliata* Fisch. protected against acetaminophen-induced liver injury in mice through scavenging free radicals and decreasing the expression of proinflammatory factors (tumor necrosis factor-*α*, nuclear factor kappa B), thereby inhibiting the inflammatory response [13].

In view of all the activities mentioned above, *V. ciliata* Fisch. has demonstrated great potential to be developed as liver protective agents. Nevertheless, to the best of our knowledge, the antioxidant activity related to scavenging superoxide and hydroxyl radicals, the hepatoprotective activity, and the mechanism of the effects of ethyl acetate extract have not been elucidated in vitro. Therefore, the aims of this study were to (1) assess the antioxidant activity of *V. ciliata* Fisch. active fragment (VCAF) and study its hepatoprotective role against t-BHP-induced injury in HepG2 cells, which is different from the previously studied drug-induced liver injury and (2) clarify the possible cellular mechanisms of hepatoprotection.

## 2. Materials and Methods

### 2.1. Reagents

Vitamin C (VC) and 1,1-diphenyl-2-picrylhydrazyl (DPPH) were purchased from Sigma-Aldrich Chemical Co. (St. Louis, MO, USA). Cell counting kit 8 (CCK-8) and caspase-3, caspase-8, and caspase-9 fluorescence metric assay kits were purchased from KeyGen BioTECH (Jiangsu, China). ALT, AST, T-AOC, SOD, DCF-DA cellular reactive oxygen species (ROS) detection assay kit, hydroxyl radical assay kit, and protein carbonyls assay kit were purchased from Nanjing Jiancheng Bioengineering Institute (Nanjing, China). The measurement kits for AChE and CAT activity were purchased from Comin Biotechnology (Suzhou, China). 8-hydroxy-2-deoxyguanosine (8-OHdG) ELISA kit was purchased from Wuhan ELISA Lab (Wuhan, China). The Annexin V-FITC and PI double staining assay kit was purchased from Vazyme Biotech (Nanjing, China). 4′,6-diamidino-2-phenylindole (DAPI) and the bicinchoninic acid (BCA) protein quantification kit were purchased from Solarbio (Beijing, China). Radioimmunoprecipitation assay (RIPA) cell lysis buffer was purchased from NCM Biotech (Suzhou, China).

### 2.2. Plant Material and Extraction

The herbs of *V. ciliata* Fisch. were purchased from Tibet Tibetan Medicine Group Co. Ltd., China. A voucher specimen (number 007211478) was identified by Dr. Jie Bai, School of Life Sciences, Sichuan University.

Approximately 100 g of the dry powder of *V. ciliata* Fisch. was weighted and extracted with 95% ethanol (1 : 5 g/mL) at 23–25°C for 24 h. The extracts were filtered, the filtrates were concentrated by using a vacuum rotary evaporator, and the concentrates were dissolved in distilled water. The solutions were sequentially extracted by petroleum ether and ethyl acetate. Lastly, the ethyl acetate extracts were evaporated to dryness at 50°C and dissolved in 50% ethanol alcohol and serum-free medium for the determination of antioxidant activity and cell experiments, respectively.

### 2.3. In Vitro Antioxidant Activity of VCAF

#### 2.3.1. Determination of Total Phenolic Content

The total phenolic contents of VCAF was determined by using the Folin-Ciocalteu method in accordance with a previously described procedure [15]. Briefly, 10 *μ*L of VCAF (0.1 mg/mL) or gallic acid (0–0.25 mg/mL) was mixed with 100 *μ*L of 10% Folin-Ciocalteu's reagent. After 5 min, the above mixture was added to 90 *μ*L of 10% Na_2_CO_3_ and then incubated for 40 min at 25°C. The absorbance was measured at 760 nm by using a microplate reader (SpectraMax M2, Molecular Devices, Sunnyvale, CA, USA). The total phenolic content was calculated from a regression equation (*y* = 3.381*x* + 0.0384, *R*^2^ = 0.9998) and expressed as mg gallic acid equivalents (GAE) per g dry extract.

#### 2.3.2. Determination of Total Flavonoid Content

The total flavonoid content of VCAF was determined based on a colorimetric method with aluminium chloride [16]. Briefly, 20 *μ*L of VCAF (0.1 mg/mL) or rutin (0–0.1 mg/mL) was diluted in 50% ethanol solution and mixed with 30 *μ*L of NaNO_2_ (5%). After 6 min, the above mixture was added to 50 *μ*L of 10% AlCl_3_; subsequently, 100 *μ*L of NaOH (1 M) was added. After 15 min, the absorbance values were measured at 510 nm and compared with 50% ethanol as a blank control. The total flavonoid content was calculated from a regression equation (*y* = 0.4196*x* + 0.0026, *R*^2^ = 0.9995) and expressed as rutin equivalents (RE) per g of dry extract.

#### 2.3.3. DPPH Radical Scavenging Assay

The assay for DPPH radical scavenging was performed with minor modifications on an improved DPPH assay [17]. Briefly, 100 *μ*L of VCAF at different concentrations (5–100 *μ*g/mL, diluted in 50% ethanol) and VC at the same concentrations were mixed with 100 *μ*L of DPPH solution (0.1 mM, in 50% ethanol). The mixed solution was allowed to stand for 30 min in the dark at 23–25°C, after which the absorbance was measured at 517 nm, with 50% ethanol as a blank control. The DPPH radical scavenging activity (%) was calculated from the following formula (Abs. = absorbance):

(1) $$\begin{array}{c} DPPH\;scavenging\;activity\left(\%\right)=\frac{Abs.50\%\;ethanol\;solution-Abs.VCAF}{Abs.50\%\;ethanol\;solution}\times 100. \end{array}$$

#### 2.3.4. Superoxide Anion Radical Scavenging Assay

The assay for superoxide anion radical scavenging was based on an improved superoxide anion radical scavenging assay [18, 19]. Briefly, 5 mL of Tris-HCl buffer (0.05 M, pH = 8.2) was mixed with 4 mL of VCAF at different concentrations (5–100 *μ*g/mL, diluted in 50% ethanol) and VC at the same concentrations after a preheating period of 20 min at 25°C. Then, 1 mL pyrogallol solution (12 mM in 1 mM HCl) was added to the solution. The mixture was immediately shaken and reacted for 5 min. Finally, 1 mL HCl (10 M) was added to terminate reaction and the absorbance values were measured at 320 nm, with 50% ethanol as a blank control. The superoxide anion radical scavenging activity (%) was calculated from the following formula (Abs. = absorbance):

(2) $$\begin{array}{c} Superoxide\;anion\;scavenging\;activity\left(\%\right)=\frac{Abs.\;50\%\;ethanol\;solution-Abs.\;VCAF}{Abs.\;50\%\;ethanol\;solution}\times 100. \end{array}$$

#### 2.3.5. Hydroxyl Radical Scavenging Assay

The hydroxyl radical scavenging assay was performed by using a commercially available detection kit in accordance with the manufacturer's instructions [20]. The absorbance values were measured at 550 nm; 50% ethanol was used as a blank control, and VC was used as a positive control. The results were expressed as U/mL.

### 2.4. Cell Culture

The human liver-derived cell line, HepG2, was obtained from American Type Culture Collection (HB-8065, VA, USA). Human normal liver cell line, HL-7702, was obtained from West China Hospital, Sichuan University. The cells were maintained in DMEM medium (Gibco BRL Co. Ltd., USA) supplemented with 10% fetal bovine serum (Gibco, Australian Origin), 100 IU/mL penicillin, and 100 IU/mL streptomycin at 37°C in an atmosphere with 5% CO_2_.

### 2.5. VCAF Toxicity Test Assay

The assessment of VCAF toxicity was performed by using cell counting kit 8 (CCK-8). HL-7702 cells in the logarithmic growth phase were seeded in 96-well plates at a density of 6 × 10^4^ cells/mL. The cells were exposed to various concentrations of VCAF for 24 h and 48 h. After the exposure period, 10 *μ*L of CCK-8 solution was added to each well and incubated at 37°C for 1 h. The optical density of each well was measured at 450 nm by using a microplate reader; complete medium used was as a blank control. Each assay was replicated in five wells, and each experiment was repeated three times.

### 2.6. Cytoprotective Effects of VCAF

HepG2 cells were seeded at a density of 5 × 10^3^ cells/well into a 96-well plate and at a density of 5 × 10^4^ cells/well into 6-well plates. When the cells reached approximately 70–80% confluence, they were treated with the indicated drugs: normal group, HepG2 cells were exposed to serum-free medium for 4 h continuously; model group, HepG2 cells were exposed to serum-free medium for 2 h and 5 mM t-BHP diluted in serum-free medium for 2 h continuously; VCAF groups, HepG2 cells were exposed to 20, 40, and 80 *μ*g/mL VCAF for 2 h and 5 mM t-BHP for 2 h continuously. The cells in the 96-well plates were used for the measurement of cell viability by using CCK-8. In the 6-well plates, the cells were washed with PBS, harvested and used for the evaluation of cellular apoptosis rate, cellular oxidative damage, and antioxidant enzyme activity. Finally, the liver marker enzymes ALT and AST were detected from the supernatant in the 6-well plates.

### 2.7. Measurement of Protein Concentration

The cell lysate proteins were quantified by using a BCA protein assay kit [21]. Briefly, after treatment with the indicated drugs, the cells were harvested and washed twice by centrifugation (Centrifuge 5424R, Eppendorf, Germany) with precooled PBS at 600 ×g for 5 min. The supernatant was discarded and the cells were resuspended in 200 *μ*L of cell lysis buffer and incubated on ice for 10 min. After a second centrifugation (8000 ×g, 5 min), the supernatants were transferred to a new centrifuge tube and placed on ice during protein level analysis using the BCA protein quantification kit. The absorbance values were measured at 562 nm by using a microplate reader. The protein concentration was calculated from a regression equation (*y* = 0.0203*x* + 0.0997, *R*^2^ = 0.9977) and expressed as mg/mL.

### 2.8. Determination of the Key Indicators Related to Oxidative Stress Response

#### 2.8.1. Determination of Intracellular ROS Levels

Intracellular ROS levels were analysed by DCFH-DA in accordance with a previous report [22]. After the HepG2 cells were treated with indicated drugs, the cells from the 6-well plates were washed twice with PBS and harvested. The cells were then labelled with 25 *μ*M DCFH-DA dissolved in PBS for 30 min in the dark and placed in 96-well black opaque plates, and the fluorescence spectrum was recorded at an excitation wavelength of 490 nm and an emission wavelength of 525 nm (SpectraMax M2, Molecular Devices, Sunnyvale, CA, USA).

#### 2.8.2. Determination of 8-OHdG Concentrations

As the most representative product of DNA oxidative damage induced by ROS, the measurement of 8-OHdG concentration was performed by using an ELISA kit in accordance with the manufacturer's instructions [23]. The absorbance values were measured at 450 nm by using a microplate reader, and the level of 8-OHdG was expressed as pg/mg protein.

#### 2.8.3. Determination of Protein Carbonyls

The determination of protein carbonyls, representative products of protein oxidative damage, was performed by using an ELISA kit in accordance with the manufacturer's instructions. The absorbance values were measured at 370 nm, and the level of protein carbonyls was expressed as nmol/mg protein.

### 2.9. Analysis of Apoptosis

#### 2.9.1. DAPI Staining

HepG2 cells were seeded at a density of 1 × 10^4^ cells/well into 24-well plates. The cells were washed twice with precooled PBS and fixed with 4% paraformaldehyde after treatment with the indicated drugs. The cells were incubated with DAPI (5 *μ*g/mL in PBS) for 5 min at 37°C, and the fluorescence was visualized under a fluorescence microscope (Olympus IX71, Japan). The cells undergoing apoptosis were identified by diminished size, plasma membrane blebbing, chromatin fragments, and condensation of the cytoplasm [24, 25].

#### 2.9.2. Flow Cytometry Apoptosis Analysis through Annexin V-FITC Staining

The apoptotic cells were identified by flow cytometry by using an Annexin V-FITC apoptosis detection kit in accordance with the manufacturer's instructions [26]. Briefly, HepG2 cells were seeded at a density of 5 × 10^4^ cells/well into 6-well plates (2 mL/well). The cells were washed twice with precooled PBS and harvested by centrifugation at 300 ×g for 5 min. The supernatant was removed, and the cells were resuspended in 200 *μ*L of Annexin V-FITC binding buffer. Then the cells were incubated with 10 *μ*L of Annexin V-FITC and 10 *μ*L of propidium iodide (PI) for 10 min at 23–25°C in the dark. Lastly, 800 *μ*L of Annexin V-FITC binding buffer was added and the apoptotic cells were analysed by flow cytometry (BD FACScalibur, USA).

### 2.10. Measurement of Caspase-3, Caspase-8, and Caspase-9

The determination of caspase-3, caspase-8, and caspase-9 activity was performed with the relevant fluorescence metric assay kit [27]. Briefly, 30 *μ*L of cell lysate protein was added to 50 *μ*L of 2 × reaction buffer and 10 *μ*L of caspase-3, caspase-8, and caspase-9 substrate. After incubation at 37°C for 1.5 h in the dark, the fluorescence spectrum was recorded at an excitation wavelength of 485 nm and an emission wavelength of 535 nm.

### 2.11. Determination of the Key Enzyme Responses to Hepatic Function

#### 2.11.1. Determinations of AST and ALT Activities

AST and ALT activities were determined by a commercial test kit with reference to the manufacturer's instructions. The absorbance values were measured at 340 nm by using a microplate reader, and the activities of AST and ALT were expressed as U/L.

#### 2.11.2. Determination of AChE Activity

AChE activity was assayed by a commercial test kit with reference to the manufacturer's instructions [28]. The absorbance values were measured at 410 nm by using a microplate reader, and the activity of AChE was expressed as nmol/min/mg protein.

### 2.12. Measurement of Antioxidase Activities

#### 2.12.1. Measurement of CAT

CAT activity was measured by a commercial test kit in accordance with the manufacturer's instructions. The absorbance values were measured at 240 nm by using a microplate reader and the activity of CAT was expressed as nmol/min/mg protein.

#### 2.12.2. Measurement of GSH

GSH activity was performed by using a commercial test kit in accordance with the manufacturer's instructions. The absorbance values were measured at 405 nm by using a microplate reader, and the activity of GSH was expressed as *μ*M/mg protein.

#### 2.12.3. Measurement of SOD

SOD activity was measured by using a commercial test kit with reference to the manufacturer's instructions. The absorbance values were measured at 450 nm by using a microplate reader and the activity of SOD was expressed as U/mg protein.

#### 2.12.4. Measurement of T-AOC

T-AOC was assayed by a commercial test kit with reference to the manufacturer's instructions [29]. The absorbance values were measured at 593 nm by using a microplate reader, and T-AOC was expressed as *μ*M/mg protein.

### 2.13. Statistical Analysis

The results were expressed as the mean ± SEM. Statistical differences in experimental data among groups were tested by one-way ANOVA (*n* = 3) (SPSS15.0, SPSS Inc., Chicago, IL, USA) and GraphPad Prism5 software (GraphPad Software, USA). Values of *p* < 0.05 were considered to be statistically significant.

## 3. Results

### 3.1. In Vitro Antioxidant Assays

Phenolic and flavonoid compounds are usually related to the antioxidant activity of medicinal plants; these compounds can act on the antioxidant defense system and alleviate oxidative stress. The higher the phenolic and flavonoid contents, the stronger the antioxidant activity [8, 11]. Our results indicated that the total flavonoid and phenolic contents of VCAF were 312.00 ± 10.00 mg/g rutin equivalent and 161.60 ± 24.33 mg/g gallic acid equivalent, respectively. These values indicated that VCAF possessed strong antioxidant activity owing to high contents of phenolic and flavonoid compounds.

**Figure 1:**
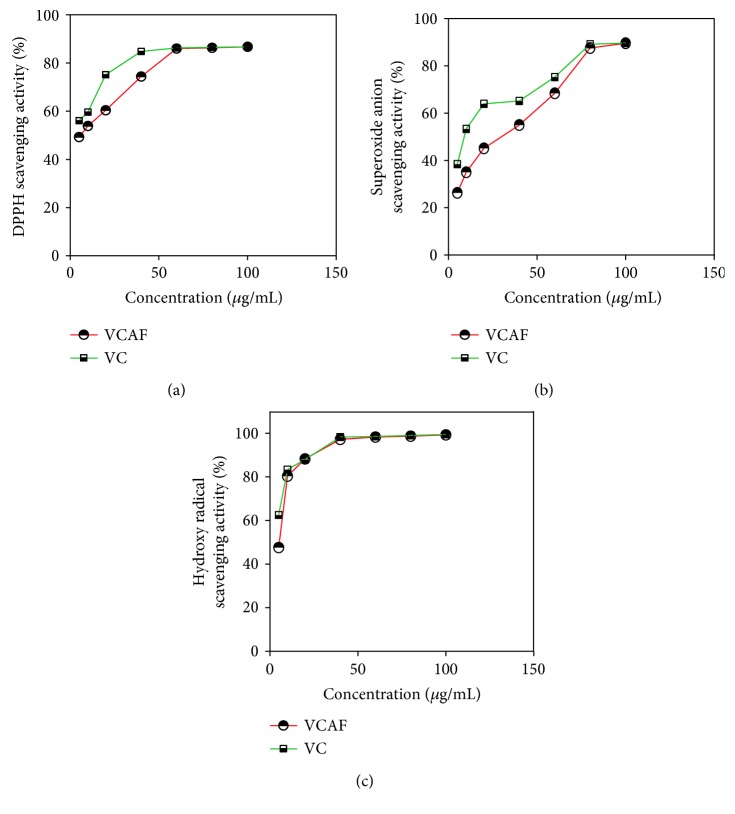
Effects of the antioxidant activity of the VCAF: (a) DPPH radical, (b) superoxide anion radical, and (c) hydroxyl radical. Data are presented as means ± SD (*n* = 3). VCAF: active fragment of *V. ciliata* Fisch.; VC: vitamin C.

Moreover, three antioxidant assays demonstrated the strong antioxidant activity of VCAF compared with VC (Figure 1). In the DPPH and hydroxyl radical scavenging assays, the IC50 values (the concentration required to scavenge 50% of the radicals) for VCAF were equivalent to VC (Table 1), which demonstrated that VCAF possessed significantly strong antioxidant activity.

### 3.2. Cytotoxicity and Cytoprotective Effects of VCAF

**Figure 2:**
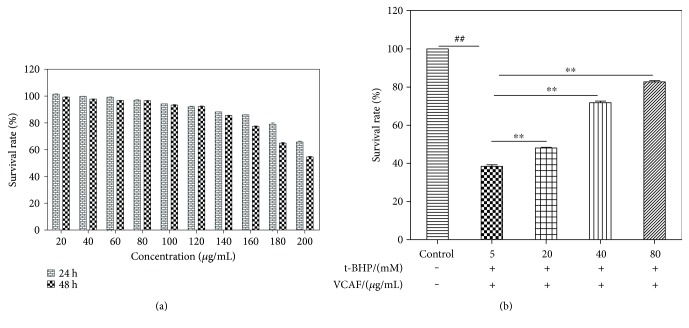
Cytotoxicity and the cytoprotective effects of VCAF. The viability of HL-7702 cells and HepG2 cells was performed by a CCK-8 assay. (a) HL-7702 cells were treated with VCAF in different concentrations (20–200 *μ*g/mL) for 24 and 48 hours. (b) HepG2 cells were pretreated for 2 h with the indicated concentrations of VCAF and then incubated for 2 h with t-BHP (5 mM). ^##^*p* < 0.01 versus control; ^∗∗^*p* < 0.01 versus 5 mM t-BHP.

No evidence of toxicity of VCAF (20–180 *μ*g/mL) was found in HL-7702 cells after treating for 24 and 48 h (Figure 2(a)). Moreover, in the HepG2, cells were exposed to t-BHP (5 mM) for 2 h after pretreatment with VCAF (20, 40, and 80 *μ*g/mL) for 2 h; we found that the viability was clearly higher than the cells that were not pretreated and that the changes were concentration dependent (Figure 2(b)).

### 3.3. Effects of VCAF on Oxidative Stress

**Figure 3:**
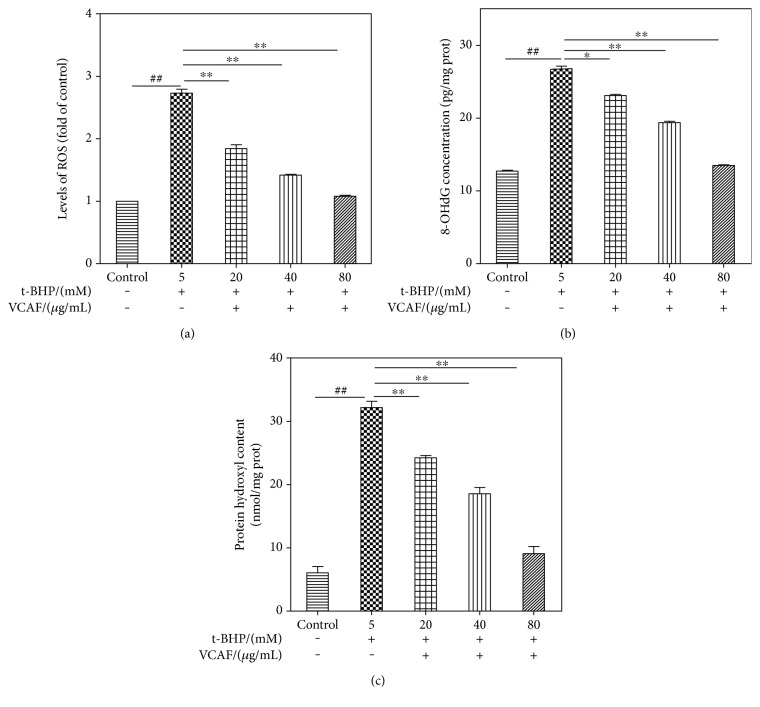
The effects of VCAF on oxidative stress injury. (a) Intracellular ROS levels. (b) Intracellular 8-OHdG concentrations. (c) Intracellular protein carbonyls content. ^##^*p* < 0.01 versus control; ^∗^*p* < 0.01 and ^∗∗^*p* < 0.01 versus 5 mM t-BHP.

The overproduction of ROS plays a crucial role in oxidative stress injury and has been confirmed to result in overloaded oxidative stress indicators, such as 8-OHdG and protein carbonyls [22]. The DCF-DA fluorescence was measured to determine intracellular ROS levels. In a concentration-dependent manner, VCAF pretreatment significantly decreased the intracellular ROS level compared with t-BHP-treated cells (Figure 3(a)). Furthermore, the levels of 8-OHdG and protein hydroxyls were determined to provide representation of the oxidatively damaged products [22]. Pretreatment with VCAF distinctly suppressed the increases in 8-OHdG and protein hydroxyl levels after t-BHP treatment in a concentration-dependent manner (Figures 3(b) and 3(c)).

### 3.4. Protective Effects of VCAF on Apoptosis

**Figure 4:**
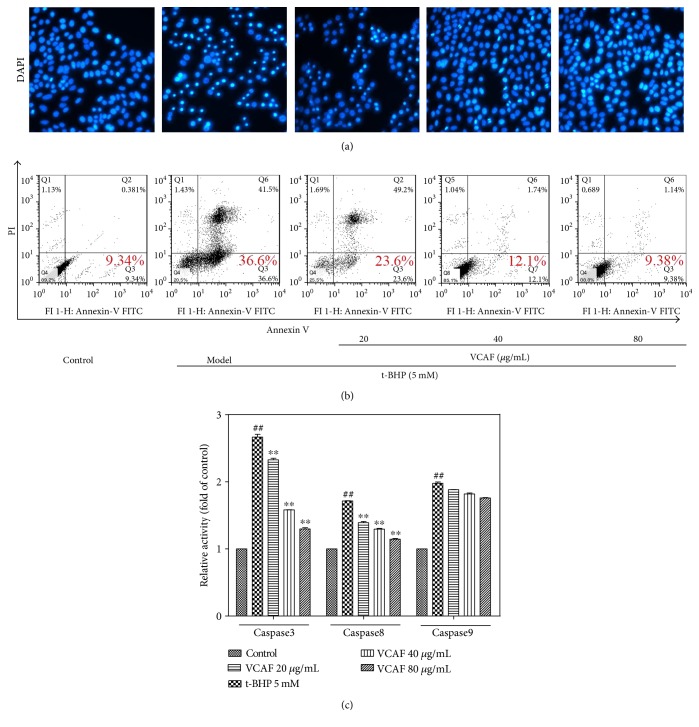
The protective effects of VCAF on apoptosis. (a) Fluorescence images (DAPI staining) of HepG2 cells at magnification of 40x. (b) Apoptosis of HepG2 cells stained with Annexin V-FITC/PI was quantified by flow cytometric analysis. (c) Analysis of the activation of caspase-3, caspase-8, and caspase-9 in the total cell lysates of HepG2 cells. ^##^*p* < 0.01 versus control; ^∗∗^*p* < 0.01 versus 5 mM t-BHP.

DAPI and Annexin V-FITC/PI staining were used to assess the effects of VCAF against t-BHP-induced apoptosis. In comparison with the untreated control cells, t-BHP induced apoptotic morphological changes, including diminished size, plasma membrane blebbing, apoptotic body formation, and condensation of the cytoplasm. In contrast, the cell morphology after VCAF pretreatment (40 and 80 *μ*g/mL) was equivalent to the control group (Figure 4(a)). Moreover, VCAF pretreatment concentration dependently suppressed the induction of early apoptosis markers after t-BHP treatment (Figure 4(b)).

### 3.5. Inhibitory Effects on Activation of Caspase-3, Caspase-8, and Caspase-9

We detected the activities of caspase-3, caspase-8, and caspase-9 as the caspase cascade is activated during apoptosis. As shown in Figure 4(c), VCAF pretreatment suppressed the increases in caspase-3 and caspase-8 compared with t-BHP treatment, but not caspase-9, which suggested that VCAF inhibited apoptosis through the downregulation of caspase-3 and caspase-8.

### 3.6. Effects of VCAF on AST, ALT, and AChE Activities

**Figure 5:**
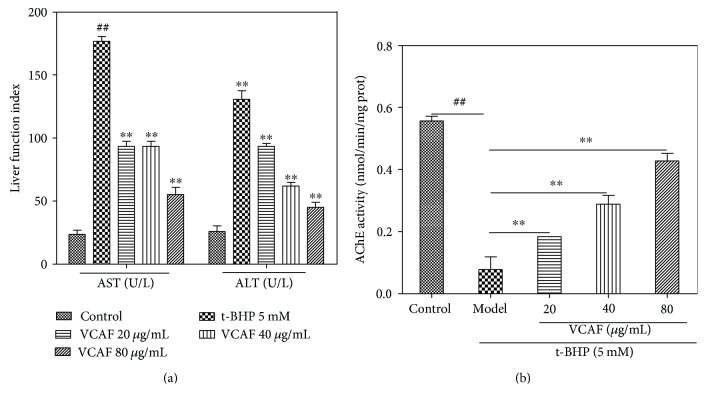
The effects of VCAF on AST, ALT, and AChE activities. (a) AST and ALT activities of cellular supernatant. (b) Intracellular AChE activities. ^##^*p* < 0.01 versus control; ^∗∗^*p* < 0.01 versus 5 mM t-BHP.

ALT, AST, and AChE are critical indices for the evaluation of liver injury. In this study, the activities of ALT and AST in the t-BHP-treated HepG2 cells were 5.2 and 7.7 times higher, respectively, than those of the corresponding control groups. However, pretreatment with VCAF distinctly suppressed the increase of ALT and AST observed in t-BHP-treated HepG2 cells in a concentration-dependent manner (Figure 5(a)). Furthermore, AChE activity showed a fivefold decrease compared with the control group after t-BHP treatment. Pretreatment with VCAF concentration dependently elevated the activity of AChE (Figure 5(b)).

### 3.7. Effects of VCAF on Antioxidase Activities

**Figure 6:**
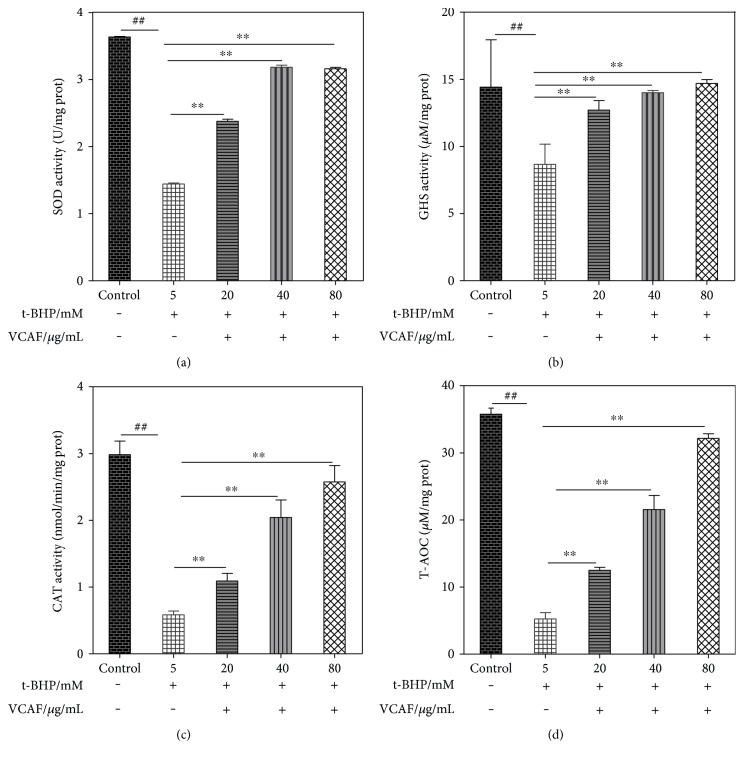
The effects of VCAF on antioxidase activities. (a) SOD activity in the supernatants of HepG2 cell lysates. (b) GSH activity in the supernatants of HepG2 cell lysates. (c) CAT activity in the supernatants of HepG2 cell lysates. (d) T-AOC in the supernatants of HepG2 cell lysates. ^##^*p* < 0.01 versus control; ^∗∗^*p* < 0.01 versus 5 mM t-BHP.

The effects of different treatments on the GSH, SOD, CAT, and T-AOC levels in the HepG2 cells are shown in Figure 6. Compared with the control group, t-BHP treatment markedly lowered the level of GSH, SOD, CAT, and T-AOC. Pretreatment with VCAF clearly prevented these decreases, which suggested that VCAF enhanced antioxidase activities and improved the antioxidant level.

## 4. Discussion

Oxidative stress caused by superfluous ROS, a key factor in the occurrence and progression of diseases such as hepatopathy, may exert oxidative damage to biomolecules such as proteins, DNA, and lipids [23, 30, 31]. Several studies have indicated that the principal sources of ROS, which are the superoxide anion, the hydroxyl radical, and H_2_O_2_, are detrimental to human health [32, 33]. Numerous physiological and biochemical processes in the human body produce oxygen free radicals and other ROS. The overproduction of free radicals and imbalanced antioxidant defense mechanisms results in apoptosis and aging [34, 35].

*V. ciliata* Fisch. is a promising candidate for a liver-protective drug on account of its considerable antioxidant and hepatoprotective activities [12]. This present study demonstrated that *V. ciliata* Fisch. possessed high phenolic and flavonoid contents and strong free radical-scavenging effects on DPPH, superoxide, and hydroxyl radicals, which implied that it could reduce the generation of ROS and relieve the damage of oxidative stress. Previous research demonstrated that VCAF and its iridoid glycoside fraction exerted hepatoprotective activity against carbon tetrachloride- and acetaminophen-induced liver injury in mice, respectively [14]. Uniformly, VCAF significantly increased the viability of HepG2 cells after exposure to t-BHP. In terms of oxidative stress, it dramatically repressed intracellular ROS generation and relieved the damage to DNA and proteins. These results suggested that VCAF enhanced antioxidant activity and protected hepatocytes against oxidative stress injury.

Apoptosis, a naturally occurring cell death process, is important for the removal of damaged and neoplastic cells [36]. However, apoptosis caused by oxidative stress is detrimental and results in adverse biological consequences [37]. VCAF suppressed the increase of early apoptosis in t-BHP-treated cells. Moreover, in the apoptosis cascades, caspase-3, caspase-8, and caspase-9 are the key proteins that trigger apoptosis, especially caspase-3 [38]. Our results revealed that VCAF inhibited apoptosis by reducing the expression of caspase-3 and caspase-8, but not caspase-9.

Liver enzymes, including ALT, AST, and AChE, reflect the health of the liver and hepatocyte integrity. Changes in these enzymes may be associated with a decrease in liver functional mass and ultimately lead to hepatopathy [39, 40]. We found that VCAF markedly reduced the activities of ALT and AST and elevated the activity of AChE; thus, VCAF was shown to improve hepatic function.

It is well known that antioxidant enzymes such as SOD, GSH, and CAT can protect against oxidative stress injury. SOD can turn O_2_^−^ into H_2_O_2_, and CAT transforms H_2_O_2_ to H_2_O. GSH is known to offer protection against chemically induced cytoxicity as a result of the elimination of reactive intermediates and hydroperoxide reduction [6, 41]. Our research found that VCAF significantly increased the activity of SOD, GSH, CAT, and T-AOC. Research indicated that VCAF can observably enhance antioxidase activities and improve antioxidant level.

**Figure 7:**
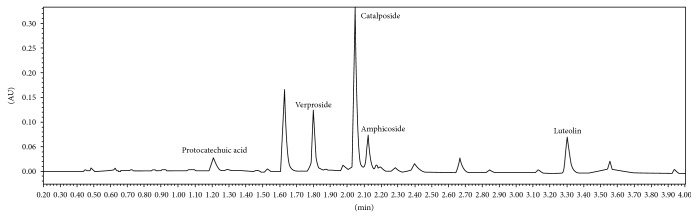
UPLC chromatograms of VCAF detected at 260 nm.

Previous studies in our laboratory have identified catalposide, verproside, luteolin amphicoside, and protocatechuic acid as the primary compounds in VCAF [11, 13] (Figure 7). These compounds have strong antioxidant, anti-inflammatory, and hepatoprotective activity, which may be related to their abundance of hydroxyl groups, as determined by pharmacological studies [42-47]. The cytoprotective effects of VCAF on HepG2 cells against t-BHP-induced oxidative stress injury can be attributed to those compounds. Moreover, the protection from catalposide, verproside, and luteolin was all related to the TNF-*α*/NF-*κ*B pathway [42, 43, 45, 46]. Therefore, it is necessary to investigate whether VCAF or its monomeric compounds (catalposide, verproside, and luteolin) protected the hepatocytes via the TNF-*α*/NF-*κ*B pathway.

## 5. Conclusion

In conclusion, VCAF has been shown to be a promising potential medicinal plant extract owing to a biomass rich in phenolic and flavonoid compounds and antioxidant activity. The cytoprotective effect of VCAF on HepG2 cells against t-BHP-induced oxidative stress injury was attributed to its ability to enhance antioxidant activity and reduce ROS production. Moreover, VCAF inhibited cellular apoptosis through the decreased expression of proapoptotic proteins, namely, caspase-3 and caspase-8. In addition, VCAF decreased t-BHP-induced oxidative stress injury in HepG2 cells through the regulation of liver enzymes and antioxidant enzyme levels. Furthermore, the cytoprotective effect of VCAF was attributed to its major compounds (catalposide, verproside, luteolin, amphicoside, and protocatechuic acid), which have been proven to be strong antioxidants with anti-inflammatory and hepatoprotective activity. Finally, our preliminary findings have proved that it is verproside and luteolin that protect HepG2 cells and rat liver BRL-3A cells against t-BHP-induced oxidative stress injury (unpublished). Further investigations will be undertaken to elucidate the hepatoprotective mechanism of verproside and luteolin.

## Figures and Tables

**Table 1:** Antioxidant activity of VCAF (IC_50_, *μ*g/mL).

Sample	IC_50_(*μ*g/mL)		
	DPPH	Superoxide anion	Hydroxyl
VCAF	6.882 ± 0.83	20.838 ± 1.319	5.021 ± 0.701
VC	3.919 ± 0.593	9.674 ± 0.986	3.620 ± 0.559
